# The Future Proofing Study: Design, methods and baseline characteristics of a prospective cohort study of the mental health of Australian adolescents

**DOI:** 10.1002/mpr.1954

**Published:** 2022-11-28

**Authors:** Aliza Werner‐Seidler, Kate Maston, Alison L. Calear, Philip J. Batterham, Mark E. Larsen, Michelle Torok, Bridianne O’Dea, Kit Huckvale, Joanne R. Beames, Lyndsay Brown, Hiroko Fujimoto, Alexandra Bartholomew, Debopriyo Bal, Susanne Schweizer, S. Rachel Skinner, Katharine Steinbeck, Julie Ratcliffe, Ju‐Lee Oei, Svetha Venkatesh, Raghu Lingam, Yael Perry, Jennifer L. Hudson, Katherine M. Boydell, Andrew Mackinnon, Helen Christensen

**Affiliations:** ^1^ Black Dog Institute University of New South Wales Sydney New South Wales Australia; ^2^ Centre for Mental Health Research The Australian National University Canberra Australian Capital Territory Australia; ^3^ Centre for Digital Transformation of Health University of Melbourne Melbourne Victoria Australia; ^4^ School of Psychology University of New South Wales Sydney New South Wales Australia; ^5^ Institute of Cognitive Neuroscience University College London London UK; ^6^ Discipline of Child and Adolescent Health Faculty of Medicine and Health The University of Sydney Sydney New South Wales Australia; ^7^ College of Nursing and Health Sciences Flinders University Adelaide South Australia Australia; ^8^ School of Women's and Children's Health University of New South Wales Sydney New South Wales Australia; ^9^ Applied Artificial Intelligence Institute Deakin University Burwood Victoria Australia; ^10^ Telethon Kids Institute University of Western Australia Perth Western Australia Australia; ^11^ Discipline of Psychiatry and Mental Health University of New South Wales Sydney New South Wales Australia

**Keywords:** adolescent development, cohort profile, developmental psychology, mental health and wellbeing, school mental health

## Abstract

**Objectives:**

The Future Proofing Study (FPS) was established to examine factors associated with the onset and course of mental health conditions during adolescence. This paper describes the design, methods, and baseline characteristics of the FPS cohort.

**Methods:**

The FPS is an Australian school‐based prospective cohort study with an embedded cluster‐randomized controlled trial examining the effects of digital prevention programs on mental health. Data sources include self‐report questionnaires, cognitive functioning, linkage to health and education records, and smartphone sensor data. Participants are assessed annually for 5 years.

**Results:**

The baseline cohort (*N* = 6388, *M* = 13.9 years) is broadly representative of the Australian adolescent population. The clinical profile of participants is comparable to other population estimates. Overall, 15.1% of the cohort met the clinical threshold for depression, 18.6% for anxiety, 31.6% for psychological distress, and 4.9% for suicidal ideation. These rates were significantly higher in adolescents who identified as female, gender diverse, sexuality diverse, or Aboriginal and/or Torres Strait Islander (all *p*s < 0.05).

**Conclusions:**

This paper provides current and comprehensive data about the status of adolescent mental health in Australia. The FPS cohort is expected to provide significant insights into the risk, protective, and mediating factors associated with development of mental health conditions during adolescence.

## 1 INTRODUCTION

The global prevalence of mental health conditions in young people is significant (Shorey et al., 2022). Approximately half of mental health conditions emerge before the age of 18 years, with peak age of onset at 14.5 years (Solmi et al., 2022). Among children and adolescents, depression and anxiety disorders are common, with global prevalence rates estimated to be up to 8% for depression (Shorey et al., 2022) and 6.5% for anxiety disorders (Polancyk et al., 2015). Evidence from Australian population surveys mirror this data, with 12‐month prevalence rates for depression and anxiety disorders estimated to be 8% and 7% respectively (Lawrence et al., 2015). There is a significant increase in the rate of mental health conditions and particularly depression with the transition from childhood to adolescence (Costello et al., 2011; Lawrence et al., 2015). Depression is the leading cause of disability in children and adolescents (Erskine et al., 2015) and both depression and anxiety disorders are major drivers of disease burden in this age group (Klaufus et al., 2022). Poor mental health is also associated with suicidality and self‐harm, both of which are significant issues for young people. According to Australian data, suicide is the leading cause of death in people aged 15–44 years (Australian Bureau of Statistics [ABS], 2020), and self‐harm rates are increasing, particularly among young women (ABS, 2022a).

While there are effective clinical treatments for depression and anxiety disorders, they are limited in availability and do not meet the current demand, particularly for young people (Lawrence et al., 2015; Slade et al., 2009). This is in part due to the stigma associated with service use, difficulties with access, and equity of care (Lawrence et al., 2015). In order to effectively address the global disease burden of mental ill‐health, there is growing recognition that prevention and early intervention approaches delivered before, or at, first onset should form part of the strategy, in addition to treatment approaches (Cuijpers et al., 2021). Research has established that prevention programs reduce symptoms and prevent incident episodes of depression and anxiety (e.g., Stockings et al., 2015; van Zoonen et al., 2014; Werner‐Seidler et al., 2021). Early intervention is critical because untreated mental illness with onset during childhood or adolescence is a key predictor of illness severity and recurrence during later adolescence (Neufeld et al., 2017) and into adulthood (Korczak & Goldstein, 2009). Therefore, delivering evidence‐based interventions early will not only reduce the duration and severity of the incident episode, but will also reduce the likelihood of future episodes later in life (Mueller et al., 1999).

Research to date has found small but consistent prevention effects for depression and anxiety interventions (Stockings et al., 2015; van Zoonen et al., 2014; Werner‐Seidler et al., 2021). These effects are likely to be small, at least in part, because contributing risk factors and trajectories of symptom development are not yet well understood (Forsman et al., 2015; Wittchen et al., 2014). An enhanced understanding of mental health risk and protective factors will lead to the development of more precise, targeted, and effective programs for prevention and early intervention. Prospective cohort studies are a key component to building this understanding.

Existing cohort studies that investigate child and adolescent mental health have provided important insights into understanding mental health risk and protection, but many have been associated with limitations. First, the large‐scale cohort studies in this area take a general focus on development and health, and accordingly, assess a broad range of outcomes for which mental health is just one, limiting the breadth, precision of measurement, and contribution of the overall study to mental health specifically (Harris, 2013; Sanson et al., 2002; Scholtens et al., 2015). For example, the Longitudinal Study of Australian Children is a study of 10,000 Australian children that collects a wide range of data including parenting, family relationships, education, and health (Sanson et al., 2002). While there is some measurement of social and emotional outcomes, these are limited in scope and do not provide the rich mental health data needed to identify mental health trajectories. A notable exception is the Adolescent Brain Cognitive Development Study in the USA, involving over 10,000 children aged 9–10 years (Garavan et al., 2018). This study has a dual focus on the neurobiological and psychological development of children through to adulthood, and includes a broad range of measures including neuroimaging, cognitive, biospecimen and individual mental and physical health factors (Karcher & Barch, 2020).

Second, of the cohort studies that do focus specifically on risk factors for adolescent mental illness, sample sizes are relatively small, ranging from 200 to 1200 participants (Beesdo‐Baum et al., 2020; De la Torre‐Luque et al., 2020; Ellis et al., 2017; Grootendorst‐van Mil et al., 2021). This limits the potential to examine risk factors associated with subgroups of adolescents, and may not accommodate for attrition rates, diminishing the potential for appropriately powered longitudinal analyses.

The Future Proofing Study (FPS) aims to overcome these limitations by utilizing large‐scale, comprehensive, and long‐term data relating to adolescent mental health, thereby advancing knowledge about the factors associated with the onset and developmental trajectories of a broad range of adolescent mental health conditions. The FPS was established as a 5‐year prospective cohort study with an embedded cluster‐randomized controlled trial (cRCT) conducted in Australian secondary schools. Full details of the trial protocol have been published elsewhere (Werner‐Seidler et al., 2020). A separate process evaluation incorporating teacher and school staff perspectives has also been conducted (Beames et al., 2021; Beames et al., submitted).

Future Proofing Study data sources include annual student self‐report questionnaires to assess mental health, wellbeing, sleep, and a wide range of other individual, environmental, and social factors. These are complemented by smartphone‐collected measures that will be examined as potential digital predictors and/or correlates of adolescent mental health and wellbeing. Smartphone‐collected measures include cognitive task performance (e.g., working memory, executive function), typing and speech characteristics, ecological momentary assessment (EMA), and passive device sensor data (e.g., Global Positioning System (GPS), accelerometry, gyroscope). Smartphone‐collected data will be monitored over time to explore associations with changes in adolescent mental health. For example, emerging research has shown that changes in activity levels as detected from smartphone sensors are associated with wellbeing, however, this has not yet been examined comprehensively (Muller et al., 2020). Modeling and machine learning approaches will be used to develop reliable and valid indicators for prediction of mental illness onset and disease trajectories (Barnett et al., 2019). Smartphone‐collected data will be examined together with other data sources to determine if superior and more accurate prediction can be achieved by combining data sources, compared to using individual measures. Ultimately, this may lead to data‐informed approaches for personalizing and enhancing intervention delivery. Finally, linkage to government records relating to health and education (e.g., emergency department attendance, hospital data from birth, academic data throughout schooling) will be utilized to provide objective information about risk factors, health outcomes, service utilization, and educational outcomes associated with adolescent mental health. To date, there have not been any mental health specific cohort studies *of this scale* which incorporate such a broad range of different data sources concurrently into the one study (Beesdo‐Baum et al., 2020; Ellis et al., 2017).

The broad questions we will address with this cohort are as follows:

(i) What is the prevalence of mental health conditions in an adolescent sample, and how do these change at an individual level over time?
(ii) What individual factors are associated with the development of, and protection against, mental health symptoms and conditions for a range of disorders (e.g., depression, anxiety, behavioral, and eating disorders), self‐harm, suicidal ideation, and suicidal behavior, cross‐sectionally and over time?
(iii) What social and environmental factors (e.g., socioeconomic status, geographical location, school factors) are associated with the development of, and protection against, mental health symptoms and conditions cross‐sectionally and over time?
(iv) Are there identifiable and distinct trajectories of mental ill‐health over time, and how can they be distinguished? Do these trajectories have different risk factors, and can these trajectories be predicted from risk factors measured at baseline or early life experience?
(v) Does passively collected smartphone data or cognitive task data correlate with or predict mental illness and wellbeing, cross‐sectionally and over time?
(vi) What is the relationship between mental health conditions and medical history, health service utilization and academic performance, as measured by linked government records, cross‐sectionally and over time?

The aim of the embedded cRCT is to investigate whether two digital prevention interventions, delivered during the first 2 years of the study, prevent or reduce depression and other mental health conditions, relative to a control group. Full details can be found in the protocol (Werner‐Seidler et al., 2020).

The purpose of the current paper is to provide a description and reference to the design, methods, and baseline characteristics of the FPS cohort.

## 2 METHOD

### 2.1 Design

The FPS is a prospective cohort study with an embedded cRCT (the trial protocol has been published elsewhere: Werner‐Seidler et al., 2020). Study variables were assessed at baseline and will be collected annually for 5 years. Ethics approvals were obtained from the University of New South Wales (NSW) Human Research Ethics Committee (HC180836), the State Education Research Applications Process for the NSW Department of Education (SERAP2019201), and relevant Catholic Schools Dioceses across Australia.

### 2.2 Setting

Enrollment in the FPS occurred in 134 secondary schools located across Australia, including government and non‐government (independent and Catholic) schools. Recruitment was conducted from March 2019 to March 2022. All NSW government and independent secondary schools and eligible NSW Catholic secondary schools were invited to participate. Independent schools in capital cities from around Australia were also invited to participate. The relative focus on NSW schools was by design, to minimize logistical barriers associated with in‐school data collection conducted by the research team. Schools were required to have a counselor, psychologist, or wellbeing staff member onsite during the data collection phase. Written informed consent was provided from a parent or guardian and the adolescent prior to study participation. Baseline data collection took place at schools across three separate Year 8 cohorts (students aged 13–14 years): the 2019 Cohort from August‐September 2019; the 2020 Cohort from August‐November 2020; the 2021 Cohort from April‐December 2021 (extended to March 2022 due to COVID‐19).

### 2.3 Participants

All adolescents enrolled in Year 8 at participating schools were invited to take part. To participate, students required a smartphone with iOS or Android operating system and an active phone number.

### 2.4 Measures

See Table 1 for the full list of measures administered. Details for measures of depression, anxiety, distress, and suicidal ideation are provided below. Details for measures of disordered eating, insomnia, sleep quality, internalizing and externalizing symptoms, wellbeing, quality of life, self‐harm, suicidal behavior, psychotic‐like symptoms, alcohol and substance use can be found in the Supplementary Materials. Other non‐clinical measures will be published separately in the future.

**TABLE 1:** Summary of primary, secondary, and additional outcome measures, potential mediating and risk factors, and data collection timepoints

Self‐report measures		Baseline	Post (Stage I)	6 months	12 months	Post (Stage II) ^a^	24 months	36 months	48 months	60 months
Participant characteristics										
Demographics	Age, sex at birth, residential location, country of birth, language spoken most at home, perceived socioeconomic status, household makeup, Aboriginal and/or Torres Strait Islander identity	✓	‐	‐	‐	‐	‐	‐	‐	‐
Height and weight	Height (cm), weight (kg)	✓	‐	‐	✓	‐	✓	✓	✓	✓
Gender identity	Current gender identity	✓	‐	‐	✓	‐	✓	✓	✓	✓
Sexuality	Sexual identity and attraction	✓	‐	‐	✓	‐	✓	✓	✓	✓
Primary and secondary outcome measures										
Depression	Patient health questionnaire: Adolescent version (PHQ‐A)	✓	✓	✓	✓	✓	✓	✓	✓	✓
Psychological distress	Distress questionnaire 5 (DQ5)	✓	✓	‐	✓	‐	✓	✓	✓	✓
Anxiety	Spence children's anxiety scale short form (CAS‐8) and CAS generalized anxiety and social phobia subscales	✓	✓	‐	✓	✓	✓	✓	✓	✓
Insomnia	Insomnia severity index (ISI)	✓	✓	‐	✓	✓	✓	✓	✓	✓
Suicidal ideation	Suicidal ideation attributes scale (SIDAS)	✓	✓	‐	✓	‐	✓	✓	✓	✓
Additional outcome measures, potential mediating variables, risk factors										
Diagnosed mental health condition	Reported diagnosed mental health condition	✓	✓	‐	✓	‐	‐	‐	✓	✓
Diagnosed disability	Reported diagnosed disability	✓	‐	‐	‐	‐	‐	‐	✓	‐
Disordered eating	Screen for disordered eating (SDE)	✓	✓	‐	✓	‐	✓	✓	✓	✓
Sleep quality	Pittsburgh sleep quality index (PSQI)	✓	✓	‐	✓	✓	✓	✓	✓	✓
Internalizing and externalizing symptoms	Strengths and difficulties questionnaire (SDQ)	✓	✓	‐	✓	‐	✓	✓	✓	✓
Wellbeing	Short‐form Warwick‐Edinburgh mental wellbeing scale (SWEMWBS)	✓	✓	‐	✓	‐	✓	✓	✓	✓
Quality of life	Child health utility 9D (CHU‐9D)	✓	✓	‐	✓	‐	✓	✓	✓	✓
Self‐harm	Self‐harm questionnaire	✓	✓	‐	✓	‐	✓	✓	✓	✓
Suicidal behavior	Youth risk behavior survey (YRBS)	✓	✓	‐	✓	‐	✓	✓	✓	✓
Psychotic‐like symptoms	Adolescent psychotic‐like symptom screener (APSS)	✓	✓	‐	✓	‐	✓	✓	✓	✓
Alcohol use	Age of onset and frequency of alcohol use	✓	✓	‐	✓	‐	✓	✓	✓	✓
Other substance use	Use of cannabis, tobacco, and other substances	✓	✓	‐	✓	‐	✓	✓	✓	✓
Hospital admissions	Reported hospital admissions for physical and mental health	✓	✓	‐	✓	‐	✓	✓	✓	✓
Childhood trauma	Behavioral risk factor surveillance system ‐ Adverse childhood experience (BRFSS‐ACE)	✓	‐	‐	‐	‐	‐	‐	✓	‐
Stressful life events	Exposure to stressful life events	‐	‐	‐	✓	‐	✓	✓	✓	✓
Personality	Big five inventory‐10 (BFI‐10)	✓	‐	‐	‐	‐	‐	‐	‐	‐
Social support	Schuster social support scale (SSSS)	✓	✓	‐	✓	‐	✓	✓	✓	✓
School connectedness	Items from programme for international student assessment (PISA)	✓	✓	‐	✓	‐	✓	✓	✓	‐
School climate	School climate measure	✓	✓	‐	✓	‐	✓	✓	✓	‐
Bullying	Frequency and type of bullying	✓	✓	‐	✓	‐	✓	✓	✓	✓
Resilience	Connor‐Davidson resilience scale (CD‐RISC‐10)	‐	‐	‐	✓	‐	✓	✓	✓	✓
Screen time	Hours of recreational screen time per day	✓	✓	‐	✓	‐	✓	✓	✓	✓
Social media use	Maladaptive Facebook use survey (MFUS), adapted to apply to social media use more broadly	✓	✓	‐	✓	‐	✓	✓	✓	✓
Pubertal development	Menarche, voice breaking and Tanner stages	✓	‐	‐	✓	‐	✓	✓	✓	✓
Romantic relationships	Reported romantic relationships	✓	‐	‐	✓	‐	✓	✓	✓	✓
Sexual behavior	Reported sexual behavior	‐	‐	‐	‐	‐	✓	‐	✓	‐
COVID‐19 exposure	Experience of COVID‐19 pandemic and child traumatic stress questionnaire (CTSQ)	✓	✓	‐	✓	‐	✓	✓	‐	‐
Bushfire exposure	Bushfire exposure questionnaire (BEQ) and child traumatic stress questionnaire (CTSQ)	✓	‐	‐	✓	‐	✓	✓	✓	✓
Flood exposure	Exposure to Australian flood disaster in 2022	‐	‐	‐	✓	‐	✓	✓	‐	‐
Top three concerns	Current top three issues of concern	✓	✓	‐	✓	‐	✓	✓	✓	✓
Priorities for the future	Social and political issues of importance to young Australians	‐	‐	‐	✓	‐	✓	✓	✓	✓

School characteristics (imputed from ACARA schools database)		Baseline	Post (stage I)	6 months	12 months	Post (stage II) ^a^	24 months	36 months	48 months	60 months
Location	State, local government area	✓	‐	‐	‐	‐	‐	‐	‐	‐
Remoteness	Accessibility and remoteness index of Australia (ARIA+)	✓	‐	‐	‐	‐	‐	‐	‐	‐
Sector and type	Government or non‐government, combined or secondary	✓	‐	‐	‐	‐	‐	‐	‐	‐
Size	Full time equivalent students, full time equivalent teaching staff	✓	‐	‐	‐	‐	‐	‐	‐	‐
Socio‐educational advantage	Index of community socio‐educational advantage (ICSEA)	✓	‐	‐	‐	‐	‐	‐	‐	‐

Smartphone‐collected data		Baseline	Post (stage I)	6 months	12 months	Post (stage II) ^a^	24 months	36 months	48 months	60 months
Self‐rated mood	Ecological momentary mood rating on 5‐point scale	✓	✓	‐	✓	‐	✓	‐	‐	‐
Affective card sorting task	Wisconsin card sorting task, with/without affective distractors	✓	✓	‐	✓	‐	✓	‐	‐	‐
Affective backward span task	Digit span backwards, with/without affective distractors	✓	✓	‐	✓	‐	✓	‐	‐	‐
Typing task	Copying and free typing about every day and affective topics	✓	✓	‐	✓	‐	✓	‐	‐	‐
Voice task	Sentence narration, phonemes, recall and free speaking on provided topics	✓	✓	‐	✓	‐	✓	‐	‐	‐
Movement (opt‐in)	Accelerometery and gyroscope device sensor data	✓	✓	‐	✓	‐	✓	‐	‐	‐
Location (opt‐in)	GPS device sensor data	✓	✓	‐	✓	‐	✓	‐	‐	‐
Data linkage (opt‐in)										
Birth and early development		Birth registrations, neonatal intensive care, perinatal care								
Health service utilization		Admission to hospital and emergency department, ambulance use, mental health ambulatory care, use of health services under Medicare and Pharmaceutical Benefits Scheme								
Physical health outcomes		Self‐harm and suicide attempt, cancer registry, notifiable conditions, death registrations								
Educational outcomes		Standardized test results from school years 3, 5, 7, 9, 10 and 12								

*Note*: References for the measures in Table 1 are available from the authors upon request.

^a^ Participants selected to take part in Stage II of the RCT only.

#### 2.4.1 Participant school and individual characteristics

Participants' school characteristics were imputed from a publicly available database curated by the Australian Curriculum, Assessment and Reporting Authority (ACARA), and included state, remoteness (e.g., major cities, inner regional, outer regional; ascertained through the Accessibility and Remoteness Index of Australia, ARIA+; Australian Institute of Health and Welfare [AIHW], 2004), school sector, school size, and Index of Community Socio‐Educational Advantage (ICSEA: an index applied to all Australian schools, calculated from parent and community socio‐demographic data; ACARA, 2020). Participants' individual characteristics were assessed via self‐report questionnaires and included age, sex at birth, gender identity, sexuality, Aboriginal and/or Torres Strait Islander identity, country of birth, language spoken most at home, household makeup, perceived socioeconomic status, and mental health and disability diagnoses.

#### 2.4.2 Depression

The Patient Health Questionnaire for Adolescents (PHQ‐A; Johnson et al., 2002) is an adaptation of the PHQ‐9, a nine‐item depression severity screening tool based on Diagnostic and Statistical Manual of Mental Disorders‐Fourth Edition criteria (range 0–27; higher score indicates greater depression). A threshold of ≥15, reflecting moderately severe symptoms, was used to determine caseness. The internal consistency of the PHQ‐A in the current study was excellent (Cronbach's *α* = 0.88).

#### 2.4.3 Anxiety

The Children's Anxiety Scale Short‐Form (CAS‐8) is an eight‐item measure of anxiety, based on the Spence children's anxiety scale (CAS; Spence, 1998). The CAS‐8 incorporates questions assessing generalized anxiety and social anxiety. The CAS‐8 has demonstrated good reliability and provides population‐level, standardized norms, with a range of 0–24 (higher score indicates greater anxiety; Spence et al., 2003; Spence et al., 2014). A threshold of ≥14 was used to determine caseness. The internal consistency of the CAS‐8 was high in the current study (*α* = 0.88). An additional 7‐items from the CAS were administered to allow for calculation of the CAS Social Phobia and Generalized Anxiety subscales (each with total scores ranging 0–18).

#### 2.4.4 Psychological distress

The Distress Questionnaire‐5 (DQ5; Batterham et al., 2016) is a five‐item screening tool assessing psychological distress. The scale has strong psychometric properties, a range of 5–25 (higher score indicates greater distress), and a threshold of ≥14 as the clinical cut‐off. The internal consistency of the DQ5 in the current study was good (*α* = 0.88).

#### 2.4.5 Suicidal ideation

The Suicidal Ideation Attributes Scale (SIDAS) is a measure of suicidal ideation severity in the past month. It has high internal consistency and good convergent validity (Van Spijker et al., 2014). The score range is 0–50, with a higher score indicating higher suicidal ideation. A threshold of ≥21 was used to determine clinical levels of suicidal ideation. Internal consistency of the SIDAS was good in the current study (*α* = 0.78).

### 2.5 Study size

The sample size was based on an estimate to detect small (*d* = 0.29) between‐group differences in the cRCT component of the study on symptoms of depression (the primary outcome), with power set at 0.80 (for details, see Werner‐Seidler et al., 2020). The target sample size was set at 10,000 participants. A cluster‐randomization approach was employed at the school level with a 1:1 allocation, projected to be 5000 participants in each arm (Werner‐Seidler et al., 2020). The intention was to use the control arm as the cohort study sample and to combine the two arms *if* the cRCT results showed no effects of the interventions on mental health.

### 2.6 Study apps

#### 2.6.1 Future Proofing app

The Future Proofing app was developed by the Black Dog Institute and Deakin University for data collection. Cognitive functioning (executive function and working memory), typing, speech, and EMA data are actively collected via app‐specific tasks. Passively collected sensor data includes location data (GPS) and movement data (accelerometery, gyroscope; Barnett et al., 2019).

#### 2.6.2 SPARX‐Future Proofing (SPARX‐FP)

SPARX‐FP is an app‐based, gamified Cognitive Behavioral Therapy (CBT) prevention program. SPARX‐FP was adapted in both format and content from a web‐based depression treatment program to an app‐based prevention program (Merry et al., 2012; Perry et al., 2017). The prevention version has been found to reduce symptoms of depression (*d* = 0.29) in school students (Perry et al., 2017).

#### 2.6.3 Sleep Ninja

Sleep Ninja is an app that delivers CBT for insomnia. Sleep Ninja has been shown to be effective in improving sleep difficulties (*d* = 0.41) and reducing symptoms of depression (*d* = 0.28) and anxiety (*d* = 0.27) in adolescents (Werner‐Seidler et al., 2019; Werner‐Seidler et al., submitted).

See protocol (Werner‐Seidler et al., 2020) for further details about these interventions and their delivery in FPS.

### 2.7 Procedure

**FIGURE 1:**
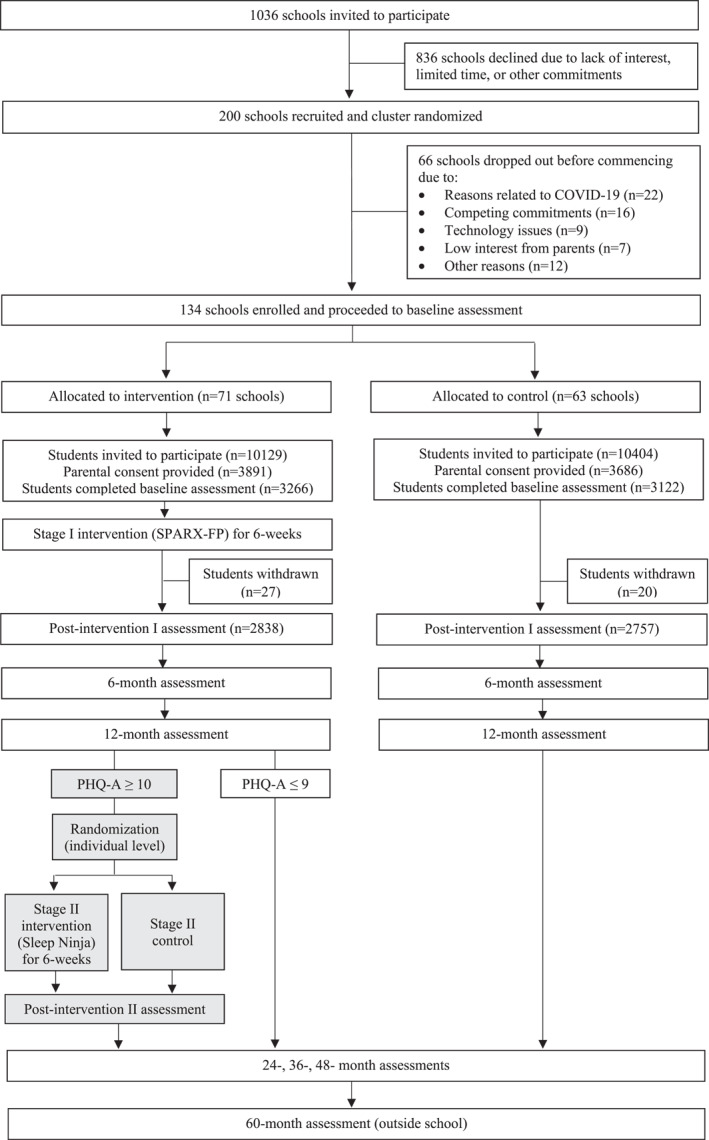
Participant flow diagram

Figure 1 shows the study timeline and flow. A total of 1036 Australian secondary schools were invited to participate in the study by letter, email, and phone. Concurrently, the study was advertised on social media and promoted at school conferences. Interested schools provided a signed letter of support from their executive and were then randomized (at the school level) using a computer‐generated randomization schedule into the intervention or control condition for the cRCT component of the study, stratified by school size, school remoteness, co‐educational versus single sex enrollment, and index of socio‐educational advantage (ICSEA). In the school term prior to study commencement, recruitment materials were provided to schools to share with Year 8 families inviting parents to provide consent for their child's participation. To minimize sampling bias, recruitment materials were produced in several languages (English, Arabic, Chinese) and parental consent was accepted online, on paper forms, or by phone. Additional opt‐in consent was obtained from parents for collection of sensor data via the Future Proofing app, and for participation in the data linkage component of the study.

On the date of the baseline assessment, participating students were invited to attend a group session at school facilitated by the study team. Students were introduced to the study and then logged in to the study website to provide individual consent and complete the baseline questionnaire on their own computer, smartphone, or tablet. They then downloaded the Future Proofing app on their smartphones to complete cognitive tasks, typing, speech, and EMA measures. All questionnaires and study tasks were completed independently, without input from the study team. Students in the intervention arm were subsequently invited to download and commence using the depression prevention program (SPARX‐FP), which they could access for 6 weeks (Stage I of the RCT). Most baseline assessments were facilitated in person by the study team (57% of all sessions). Assessments that coincided with physical distancing restrictions imposed due to COVID‐19 were facilitated remotely over Zoom, with students joining either from school (41% of all sessions) or home (2% of all sessions). At 12 months (Stage II of the RCT), students in the intervention arm with moderate to high levels of depression were independently re‐randomized to either the Sleep Ninja intervention or a control group. Those who received the Sleep Ninja intervention had access to the program for 6 weeks.

Follow‐up assessments are conducted primarily at school to minimize loss at follow‐up, and are scheduled for 6‐weeks, 6‐months, 12‐months, 24‐months, 36‐months, 48‐months, and 60‐months post‐baseline. At the 6‐week assessment, all students received a $20 gift card to reimburse personal data costs. Linkage with government records relating to health and educational outcomes will be conducted in 2023 and 2026.

### 2.8 Statistical analysis

Data quality was routinely monitored by the study Data Manager. Data were automatically collected using a bespoke study‐specific platform, hosted on the Black Dog Institute Research Engine. Data from the Research Engine were exported to Excel for preparation and then imported to SPSS Statistics v28 for analyses.

The focus of this paper is to present characteristics of the cohort and their mental health symptoms at baseline, using descriptive and frequency statistics. Data are presented overall and segmented by key demographic characteristics known to be associated with mental health conditions (ABS, 2022a; Lawrence et al., 2015). An analysis of variance approach was used to investigate gender differences in baseline mental health symptoms, wellbeing, and quality of life, using Bonferroni adjustments. Mental health symptom profiles for primary and secondary outcomes are presented, together with the proportion of participants above scale specified clinical cut‐offs. Chi‐square analyses were used to compare participants based on their school remoteness and ICSEA, and for participants who were gender or sexuality diverse, Aboriginal and/or Torres Strait Islander, or spoke English as a second language, and to compare these subgroups on mental health symptoms. Reported effect sizes are partial eta squared (*η*_*p*_^2^) for continuous outcomes where 0.01 is small, 0.06 is medium and 0.14 is large, and phi (*φ*) for categorical outcomes where 0.10 is small, 0.30 is medium and 0.50 is large (Cohen, 1988; Keppel, 1991).

## 3 RESULTS

A total of 200 schools consented to participate and were randomized. However, as baseline data collection coincided with multiple COVID‐19 lockdowns, a total of 66 schools withdrew from the study (see Figure 1 for reasons). There were 134 schools enrolled in the final sample, of which 77 (57.5%) were government schools and 57 (42.5%) were non‐government schools. From these 134 schools, 20,533 Year 8 students were invited to participate. Consent was obtained from 7577 parents, and baseline data were collected from a total of 6388 students. There were 3266 students randomized to the intervention arm, and 3122 students randomized to the control arm.

### 3.1 Participant school and individual characteristics

Table 2 shows participant school and individual characteristics. Table 3 shows a comparison of the key demographics from our cohort with youth population estimates in Australia.

**TABLE 2:** 

School characteristics	
School state, *n* (%)	
New South Wales	5465 (85.6)
Victoria	455 (7.1)
Queensland	196 (3.1)
Western Australia	183 (2.9)
South Australia	89 (1.4)
School remoteness, *n* (%)	
Major cities	4855 (76.0)
Inner regional	1409 (22.1)
Outer regional	124 (1.9)
School sector, *n* (%)	
Government school	3252 (50.9)
Non‐government school	3136 (49.1)
School size, mean (SD)	
FTE students	952 (279)
FTE teaching staff	76 (25)
ICSEA, median (SD)	1048 (79.6)
Individual characteristics	
Age, mean (SD)	13.90 (0.51)
Sex at birth, *n* (%)	
Female	3329 (52.1)
Male	2970 (46.5)
Not sure	33 (0.5)
Another term	<5 (<1)
Prefer not to say	52 (0.8)
Gender identity, *n* (%)	
Female	3122 (48.9)
Male	2973 (46.5)
Nonbinary	117 (1.8)
Other	67 (1.0)
Prefer not to say	109 (1.7)
Gender diversity, *n* (%)	
Cisgender	6019 (94.2)
Gender diverse	202 (3.2)
Mischievous or uninterpretable response	37 (0.6)
Prefer not to say	130 (2.0)
Sexuality, *n* (%)	
Heterosexual	4471 (73)
Gay or lesbian	103 (1.7)
Bisexual	414 (6.8)
Pansexual	119 (1.9)
Asexual	67 (1.1)
Other	96 (1.6)
Not sure	561 (9.2)
Prefer not to say	296 (4.8)
Sexuality diverse	764 (12.5)
Aboriginal and/or Torres Strait Islander identity, *n* (%)	
Not Aboriginal and/or Torres Strait Islander	5899 (92.3)
Aboriginal and/or Torres Strait Islander	338 (5.3)
Prefer not to say	151 (2.4)
Country of birth, *n* (%)	
Australia	5842 (91.5)
Others	545 (8.5)
Language spoken most at home, *n* (%)	
English	5982 (93.7)
Others	405 (6.3)
Household makeup, *n* (%)	
Two parents	5009 (78.4)
Stepfamily	648 (10.1)
Single parent	669 (10.5)
Other	62 (1.0)
Perceived socioeconomic status, *n* (%)	
High	2866 (44.9)
Medium	2083 (32.6)
Low	529 (8.3)
Prefer not to say	910 (14.2)
Mental health diagnosis, *n* (%)	
Major depression	239 (3.7)
Social anxiety disorder	383 (6.0)
Generalized anxiety disorder	501 (7.8)
Obsessive compulsive disorder	85 (1.3)
Panic disorder	120 (1.9)
Separation anxiety disorder	97 (1.5)
Alcohol use disorder	8 (0.1)
Substance use disorder	10 (0.2)
Attention deficit hyperactivity disorder	395 (6.2)
Post‐traumatic stress disorder	88 (1.4)
Schizophrenia/Psychosis	16 (0.3)
No diagnosis	5253 (82.2)
Disability diagnosis, *n* (%)	
Autism	170 (2.7)
Intellectual disability	29 (0.5)
Learning disability	135 (2.1)
Tourette syndrome	41 (0.6)
Cerebral palsy	9 (0.1)
Brain injury	16 (0.3)
Neurological disability	57 (0.9)
Hearing impairment	90 (1.4)
Visual impairment	376 (5.9)
No diagnosis	5591 (87.5)

*Note*: *N* for each measure = 6388 except sexuality (*N* = 6127), country of birth (*N* = 6387), and language spoken most at home (*N* = 6387).

Abbreviations: FTE, Full time equivalent; Gender diverse, gender identity incongruent with sex at birth (includes nonbinary gender); ICSEA, Index of Community Socio‐Educational Advantage; Sexuality diverse, gay or lesbian, bisexual, pansexual, asexual, and free text responses to ‘other’ where valid terms describing non‐heterosexual identity were provided. To protect the identities of individuals, values less than 5 are censored and reported as ‘<5’.

**TABLE 3:** Comparison of Future Proofing Study (FPS) cohort to Australian youth population estimates

	FPS Cohort (%)	Australian Population (%)
Female	48.9	48.6^a^
Born in Australia	91.5	84.0^b^
Reside in major cities	76.0	75.0^c^
Aboriginal and/or Torres Strait Islander	5.3	5.6^d^
Sexuality diverse	12.5	12.2^e^–21.1^f^
Gender diverse	3.2	2.3^f^–3.7^g^
Attends government secondary school	50.9	58.9^h^

^a^ Australian Census, reference sample 10–14 years (ABS, 2022e).

^b^ Australian Census, reference sample 10–14 years (ABS, 2022d).

^c^ Australian Institute of Health and Welfare, reference sample 15–24 years (AIHW, 2021).

^d^ Australian Census, reference sample 5–14 years (ABS, 2022c).

^e^ Victorian Agency for Health Information, reference sample 18–24 years (VAHI, 2020).

^f^ Australian Research Centre in Sex, Health and Society, reference sample 15–18 years (Fisher et al., 2019).

^g^ Mission Australia Youth Survey Report, reference sample 15–19 years (Mission Australia, 2021).

^h^ Australian Curriculum, Assessment and Reporting Authority, reference sample 12–18 years (ACARA, 2021).

Study participants were in Year 8 (*M* = 13.9 years; *SD* = 0.51). Most students (85.6%) attended schools located in NSW, the same state as the study team. Approximately three quarters of students attended schools in major cities and 24% attended schools located in inner and outer regional areas, reflecting the spread of the Australian population (AIHW, 2021). Approximately half of participating students attended government secondary schools (50.9%), which is slightly lower than the proportion of students attending government secondary schools in the Australian population (58.9%; ACARA, 2021). Schools attended by study participants had a mean number of 952 students and 76 full‐time equivalent teaching staff, with a student to teaching staff ratio of 12.5 students to one teacher, similar to the national average (ABS, 2022b). Participants attended schools with a median ICSEA of 1048, which is slightly higher than the Australian population median of 1000 (ACARA, 2020).

Participants mostly identified as female (48.9%) or male (46.5%), with 4.5% indicating that they were nonbinary, other, or preferred not to say. Most participants were cisgender (94.2%) and 3.2% were gender diverse (participants whose gender identity was incongruent with their sex at birth). In terms of sexuality, 73% indicated heterosexual identity, 12.5% indicated either ‘gay or lesbian’, ‘bisexual’, ‘pansexual’, ‘asexual’, or provided a free text response to ‘other’ describing non‐heterosexual identity using another term, 9.2% indicated ‘not sure’, and 4.8% preferred not to say. Although there is limited Australian data regarding LGBTQA + identity in young adolescents, our cohort were comparable to Australian estimates for people aged 15–24 years (Fisher et al., 2019; Victorian Agency for Health Information [VAHI], 2020; Mission Australia, 2021). A small proportion of the cohort (5.3%) identified as Aboriginal and/or Torres Strait Islander, representative of recent census estimates (ABS, 2022c). Participants were predominantly born in Australia (91.5%) and spoke mostly English at home (93.7%), higher than recent census estimates suggesting that 84% of children aged 10–14 years were Australian born (ABS, 2022d). Approximately 20% of participants lived with a stepfamily or a single‐parent family, with the majority (78.4%) in two‐parent households.

Participants provided self‐report responses to questions about lifetime mental health or disability diagnosis given by a health professional. In line with population prevalence estimates (Lawrence et al., 2015), most participants reported never having received a diagnosis of a mental health condition (82.2%) or a disability (87.5%). A depression diagnosis was reported by 3.7% of participants, which was slightly lower than the 5% prevalence level reported by young people (11–17 years) in the most recent Australian population mental health survey (Lawrence et al., 2015). For anxiety disorders, Generalized Anxiety Disorder and Social Anxiety Disorder were most common (7.8% and 6%, respectively). A diagnosis of Attention Deficit Hyperactivity Disorder was reported by 6.2% of the cohort. The most common disabilities were visual impairment (5.9%), autism (2.7%) and learning disability (2.1%; see Table 2).

### 3.2 Participant symptoms on clinical measures, wellbeing and quality of life

Participant symptom data are presented in Table 4. Data are presented overall and separated by gender identity (male, female, nonbinary). Mental health symptom levels differed significantly as a function of gender on all variables, all *p*s < 0.001 (depression, *η*_*p*_^2^ = 0.10; anxiety, *η*_*p*_^2^ = 0.17; psychological distress, *η*_*p*_^2^ = 0.15; suicidal ideation, *η*_*p*_^2^ = 0.05; disordered eating, *η*_*p*_^2^ = 0.14; insomnia, *η*_*p*_^2^ = 0.07; sleep quality, *η*_*p*_^2^ = 0.06; externalizing symptoms, *η*_*p*_^2^ = 0.03; internalizing symptoms, *η*_*p*_^2^ = 0.12), with female gender being associated with higher symptom levels relative to male gender (all *p*s < 0.001). This pattern was replicated for wellbeing (*η*_*p*_^2^ = 0.04) and quality of life (*η*_*p*_^2^ = 0.12), with female gender being associated with lower levels of wellbeing and quality of life, relative to males (*p*s < 0.001). Adolescents who identified as nonbinary had significantly higher symptoms relative to males and females across all variables (*p*s < 0.001), except for disordered eating, where there was no difference between those who identified as nonbinary and females (*p* = 0.07).

**TABLE 4:** Symptoms on clinical measures, wellbeing, and quality of life in overall cohort and in male, female, and nonbinary participants

	Total	Male	Female	Nonbinary
	*N* = 6388	*n* = 2973	*n* = 3122	*n* = 117
PHQ‐A	7.36 (6.3)	5.4 (5.3)	8.58 (6.3)	15.9 (7.1)
CAS‐8	8.26 (5.5)	5.84 (4.5)	10.11 (5.3)	13.82 (5.9
CAS‐SP	7.21 (4.6)	5.17 (3.8)	8.81 (4.3)	11.41 (4.7)
CAS‐GAD	6.51 (4.5)	4.5 (3.6)	8.1 (4.4)	10.56 (4.5)
DQ5	11.34 (5.1)	9.33 (4.2)	12.76 (5)	17.54 (5)
SIDAS	2.84 (7.5)	1.6 (5.4)	3.49 (8.3)	12.45 (13.8)
SDE	1.48 (1.5)	0.9 (1.2)	1.96 (1.5)	2.28 (1.5)
ISI	7.06 (5.5)	5.61 (4.9)	8.00 (5.6)	13.53 (6.5)
PSQI	6.12 (3.7)	5.33 (3.5)	6.55 (3.6)	10.38 (4)
SDQ‐total	12.72 (6.9)	10.63 (6.2)	14.05 (6.9)	20.72 (6.6)
SDQ‐internalizing	5.9 (3.94)	4.48 (3.46)	6.89 (3.82)	10.27 (3.93)
SDQ‐externalizing	6.81 (4.0)	6.15 (3.76)	7.16 (4.08)	10.46 (3.58)
SWEMWBS	21.2 (4.9)	22.2 (5.2)	20.6 (4.4)	17.3 (4.3)
CHU‐9D	0.65 (0.26)	0.75 (0.23)	0.59 (0.26)	0.38 (0.24)

*Note*: Data are mean (SD). Higher scores reflect greater levels of the variable being measured.

Abbreviations: CAS‐8, Children's Anxiety Scale Short Form‐8 (Anxiety); CAS‐GAD, Spence Children's Anxiety Scale—Generalized Anxiety Disorder subscale; CAS‐SP, Spence Children's Anxiety Scale—Social Phobia subscale; CHU‐9D, Child Health Utility 9D (Quality of Life); DQ5, Distress Questionnaire 5 (Psychological Distress); ISI, Insomnia Severity Index; PHQ‐A, Patient Health Questionnaire for Adolescents (Depression); PSQI, Pittsburgh Sleep Quality Index; SDE, Screen for Disordered Eating; SDQ‐externalizing, Strengths and Difficulties Questionnaire—Externalizing subscale; SDQ‐internalizing, Strengths and Difficulties Questionnaire—Internalizing subscale; SDQ‐total, Strengths and Difficulties Questionnaire—Total Difficulties Score; SIDAS, Suicidal Ideation Attributes Scale; SWEMWBS, Short Warwick‐Edinburgh Mental Wellbeing Scale. Data are presented by gender identity.

Participant data were collected from 2019 to 2022 so we conducted ANOVAs on the outcomes reported in Table 4 with baseline year entered as a fixed factor. The overall tests were significant, suggesting higher levels of mental health symptoms in later years. However, the effect sizes were all *η*_*p*_^2^<0.003 demonstrating negligible effects as a function of baseline year.

The proportion of participants meeting clinical thresholds on primary and secondary measures (depression, anxiety, psychological distress, suicidal ideation) are presented in Table 5. Data are presented overall and broken down by school remoteness, school ICSEA, gender identity, gender diversity, sexuality diversity, Aboriginal and/or Torres Strait Islander identity, and English as a second language. Participants who attended school in regional areas were more likely to meet the clinical threshold for depression (*p* = 0.046, *φ* = 0.03) and anxiety (*p* = 0.002, *φ* = 0.04) relative to participants who attended school in major cities but did not significantly differ in symptom profile for psychological distress (*p* = 0.41) or suicidal ideation (*p* = 0.52). Participants who attended less advantaged schools (ICSEA≤1000) were significantly more likely to reach the clinical threshold for depression (*p* = 0.001, *φ* = −0.04), anxiety (*p* < 0.000, *φ* = −0.06) and psychological distress (*p* = 0.017, *φ* = −0.03) relative to participants who attended more advantaged schools (ICSEA≥1001) but did not significantly differ on suicidal ideation (*p* = 0.26). Gender diverse participants were significantly more likely to reach the clinical threshold for depression (*φ* = 0.23), anxiety (*φ* = 0.17), psychological distress (*φ* = 0.18), and suicidal ideation (*φ* = 0.19) relative to cisgender participants (all *p*s < 0.0001). Similarly, sexuality diverse participants were significantly more likely to reach the clinical threshold for depression (*φ* = 0.33), anxiety (*φ* = 0.26), psychological distress (*φ* = 0.32), and suicidal ideation (*φ* = 0.23) relative to those who were heterosexual (all *p*s < 0.0001). Participants who identified as Aboriginal and/or Torres Strait Islander were significantly more likely to meet the clinical threshold for depression (*p* = 0.001, *φ* = 0.04), anxiety (*p* = 0.028, *φ* = 0.03), psychological distress (*p* = 0.014, *φ* = 0.03) and suicidal ideation (*p* < 0.000, *φ* = 0.05) relative to participants who did not identify as Aboriginal and/or Torres Strait Islander. There were no significant differences in the symptom profile of participants with English as a second language for depression, psychological distress, or suicidal ideation (all *p*s > 0.05) relative to participants who spoke mostly English at home, and participants with English as a second language were significantly less likely to meet the clinical threshold for anxiety (*p* = 0.032, *φ* = −0.03).

**TABLE 5:** Proportion of cohort meeting clinical cut‐offs for depression, anxiety, psychological distress, and suicidal ideation overall and by demographic characteristics

	PHQ‐A (%)	CAS‐8 (%)	DQ5 (%)	SIDAS (%)
Total	15.1	18.6	31.6	4.9
Major cities	14.6	17.7	31.3	4.8
Regional areas	16.7	21.2	32.4	5.2
ICSEA ≥ 1001	14.1	17.0	30.7	4.7
ICSEA ≤ 1000	17.5	22.3	33.7	5.4
Male	7.6	6.9	16.6	2.4
Female	19.1	26.9	42.0	6.1
Gender diverse	58.9	53.5	76.2	28.7
Sexuality diverse	43.5	43.1	66.5	17.7
Aboriginal and/or Torres Strait Islander	21.0	23.1	37.6	9.5
English is second language	15.8	14.6	32.6	5.3

*Note*: Data are percentages of the cohort meeting clinical cut‐offs across the PHQ‐A (depression), CAS‐8 (anxiety), DQ5 (psychological distress) and SIDAS (suicidal ideation). Gender diverse, gender identity incongruent with sex at birth (includes nonbinary gender); Sexuality diverse, gay or lesbian, bisexual, pansexual, asexual, and free text responses to ‘other’ where valid terms describing non‐heterosexual identity were provided.

Abbreviations: CAS‐8, children'’s anxiety scale; DQ5, distress questionnaire‐5; ICSEA, Index of Community Socio‐Educational Advantage; PHQ‐A, Patient Health Questionnaire for Adolescents; SIDAS, Suicidal Ideation Attributes Scale.

### 3.3 Additional variables

See Table 6 for descriptive data on self‐harm, suicidal behavior, psychotic‐like symptoms, alcohol and substance use. Data are presented overall and separated by gender identity (male, female, nonbinary).

**TABLE 6:** Self‐harm, suicidal behavior, psychotic‐like symptoms, alcohol and substance use in overall cohort and in male, female, and nonbinary participants

	Total	Male	Female	Nonbinary
Ever self‐harmed, *n* (%)	*N* = 6373	*n* = 2963	*n* = 3118	*n* = 117
Never	4966 (77.9)	2528 (85.3)	2311 (74.1)	48 (41.0)
Once	654 (10.3)	244 (8.2)	372 (11.9)	14 (12.0)
2, 3 or 4 times	403 (6.3)	115 (3.9)	241 (7.7)	24 (20.5)
5 or more times	350 (5.5)	76 (2.6)	194 (6.2)	31 (26.5)
Suicidal behavior in past 12 months, *n* (%)	*N* = 6370	*n* = 2960	*n* = 3118	*n* = 117
Suicidal ideation	499 (7.8)	159 (5.4)	277 (8.9)	29 (24.8)
Suicide plan	413 (6.5)	135 (4.6)	230 (7.4)	27 (23.1)
Suicide attempt	307 (4.8)	99 (3.3)	169 (5.4)	19 (16.9)
Psychotic‐like symptoms, *n* (%)	*N* = 6163	*n* = 2832	*n* = 3047	*n* = 114
Paranoia	778 (12.8)	176 (6.2)	516 (16.9)	45 (39.5)
Auditory hallucinations	1039 (16.9)	317 (11.2)	607 (19.9)	53 (46.5)
Visual hallucinations	808 (13.1)	276 (9.7)	449 (14.7)	33 (28.9)
Alcohol use, *n* (%)	*n* = 6161	*n* = 2831	*n* = 3046	*n* = 114
Ever had standard drink	860 (14.0)	427 (15.1)	381 (12.5)	24 (21.1)
Standard drink in past 6 months	634 (10.3)	313 (11.0)	289 (9.5)	13 (11.4)
Ever had 5+ standard drinks	211 (3.4)	108 (3.8)	93 (3.1)	<5 (<5)
Substance use in past 6 months, *n* (%)	*N* = 6156	*n* = 2830	*n* = 3042	*n* = 114
No drug use	5333 (86.6)	2430 (85.9)	2664 (87.6)	98 (86.0)
Cannabis	140 (2.3)	104 (3.7)	83 (2.7)	9 (7.9)
Tobacco	215 (3.5)	104 (3.7)	88 (2.9)	10 (8.8)
Amphetamines	18 (0.3)	9 (0.3)	<5 (<1)	<5 (<5)
Ecstasy	33 (0.5)	14 (0.5)	13 (0.4)	<5 (<5)
Hallucinogens	30 (0.5)	13 (0.5)	12 (0.4)	<5 (<5)
Sedatives	26 (0.4)	11 (0.4)	12 (0.4)	<5 (<5)
Inhalants	45 (0.7)	20 (0.7)	20 (0.7)	<5 (<5)
Other substance/s	249 (4.0)	93 (3.3)	139 (4.6)	7 (6.1)

*Note*: Data are *n* and %. To protect the identities of individuals, values less than 5 are censored and reported as ‘<5'. Data are presented by gender identity.

## 4 DISCUSSION

The FPS cohort at baseline provides the most comprehensive, up‐to‐date Australian information about mental health during the early adolescent years since the last national survey, conducted almost a decade ago (Lawrence et al., 2015). Participant characteristics reported in this paper indicate that the FPS cohort is comparable to the Australian adolescent population in relation to gender, remoteness, Aboriginal and/or Torres Strait Islander identity, and LGBTQA + identity (see Table 3 for reference samples). To the best of our knowledge, this cohort provides the most comprehensive gender diversity prevalence data for adolescents under the age of 15 in Australia, something that has been omitted from previous studies, and similarly, furthers the field by providing detailed, large‐scale prevalence data about sexuality diversity for adolescents under 15 (Fisher et al., 2019; Mission Australia, 2021; VAHI, 2020).

The FPS cohort includes a relatively high proportion of Australian‐born adolescents who report speaking mostly English at home. It is well‐documented that culturally and linguistically diverse populations are underrepresented in health research for a range of reasons (Hughson et al., 2016; Smith et al., 2018). In the case of mental health research, these reasons may include language and communication barriers, as well as the stigma associated with accessing mental health services (Wohler & Dantas, 2017). The FPS cohort also includes more students who attend non‐government schools with a higher median ICSEA relative to students in the overall Australian population. Despite our best attempts to attract a representative sample by advertising widely, approaching all school types, and producing recruitment materials in multiple languages and formats, there is likely to be some degree of sampling bias due to the *opt‐in* nature of the study. Government schools represent 57.5% of the schools participating in the study, however, the opt‐in consent rate was higher from parents at non‐government schools. Having resources available at schools to devote to study recruitment, as well as household factors and parental availability, likely contributed to these differential consent rates, which is consistent with perspectives provided by school staff on this issue (Beames et al., submitted). Related to this point is that smartphone ownership was necessary for study inclusion, raising the possibility of sampling bias. At study commencement, estimates suggest 90% of Australian teenagers owned a smartphone (Nielsen, 2019), providing some parameters around the extent of this sampling bias risk.

Key findings from our initial baseline analyses showed that female and nonbinary adolescents had elevated symptom levels across all clinical measures relative to their male counterparts with effect sizes ranging from small (e.g., externalizing symptoms) to large (e.g., depression, anxiety, distress), with nonbinary participants faring worse than their female peers. Participants attending school in regional areas were more likely to reach clinical thresholds for depression and anxiety, and those attending less advantaged schools were more likely to report symptoms above clinical thresholds for depression, anxiety, and psychological distress, although the size of these effects for both variables were negligible. These results are in line with national data indicating that despite increased risk factors outside of metropolitan areas, prevalence rates for common mental illnesses do not differ between those living in metropolitan and regional areas of Australia (ABS, 2008). Members of minority and marginalized groups, including Aboriginal and/or Torres Strait Islander people and adolescents who were gender and/or sexuality diverse, were significantly more likely to report symptoms above clinical thresholds for depression, anxiety, psychological distress, and suicidal ideation. These effect sizes were in the small‐to‐medium range for gender and sexuality diversity and very small for Aboriginal and/or Torres Strait Islander identity, and align with existing estimates showing elevated mental health symptom levels among these groups (ABS, 2019; Russell & Fish, 2016; Strauss et al., 2020; VAHI, 2020). The FPS data extends what is known about young people by providing a reliable, population‐level estimate of the degree to which these groups show heightened symptoms, during the critical developmental phase of early adolescence.

The main strengths of the data reported in this study include the comprehensiveness of the self‐report information across a range of mental health and demographic domains, the representativeness of the sample, and the reporting of current prevalence estimates for common mental health symptoms and suicidality among Australian adolescents. There were significant mental health symptom levels reported in this cohort, with higher rates for some population groups (e.g., gender and sexuality diverse adolescents). The FPS will continue to monitor prevalence levels in these high‐risk groups, as well as the whole population, and will provide evidence about how mental health morbidity changes over time in adolescents.

The FPS cohort will advance knowledge by leveraging a broad range of data sources (e.g., self‐report, cognitive task performance, passively collected sensor data, government health and education records) to create a rich and detailed picture of the development of adolescent mental health conditions. Moreover, the large FPS sample will provide sound statistical power for future studies to examine predictors and mediators of mental health outcomes, and the ability to use advanced statistical approaches (e.g., latent class analysis, growth curve modeling) to identify subgroups of adolescents and individual trajectories of mental health and wellbeing. Data from the FPS cohort are likely to inform the targets of prevention and early intervention strategies well into the future, providing focus for more personalized and precision‐based approaches to mental health. While not the focus of the current paper, the embedded cRCT is one of the largest of its kind and will be able to answer questions about the effectiveness of digital prevention approaches, at scale (Werner‐Seidler et al., 2020).

There are several limitations associated with the FPS that need to be considered. First, using an opt‐in school, parent, and participant consent process likely led to some sampling biases which included a slightly more advantaged and less linguistically diverse sample relative to the general Australian population. Nonetheless, the study still reflects the Australian population with respect to gender, remoteness, and other diversity characteristics. Relatedly, adolescents who were not in formal schooling were not included in this cohort study. Second, by coincidence, baseline data were collected during 2019–2022. From 2020 to 2021, many regions of Australia were subject to varying levels of COVID‐19 related lockdowns and restrictions. It is within this context that the baseline data need to be considered. While a potential limitation given this broader context, this timeframe provides a unique opportunity to assess how young people fare over the long term after such significant disruption. Finally, the embedded cRCT may be a limitation of the cohort study *if* intervention effects on mental health outcomes are detected. If this is the case, the control arm (*n* = 3122) will comprise the sample to answer our core cohort study research questions. Although a limitation to the pure cohort study, if effects of the interventions are detected, there will be an opportunity to examine potential interaction of risk and protective factors on intervention effects.

The FPS cohort is expected to provide a major contribution to understanding different aspects of development, experience, and behavior as risk and/or protective factors for mental ill‐health and wellbeing during the adolescent years.

## CONFLICT OF INTEREST

The authors have no conflicts of interest to declare.

## Supporting information

Supplementary Material

## Data Availability

Data available on request due to privacy/ethical restrictions.
