# Supplementary material for: The Future Proofing Study: Design, methods and baseline characteristics of a prospective cohort study of the mental health of Australian adolescents
Source: Int J Methods Psychiatr Res. 2022 Nov 28;32(3):e1954. doi: 10.1002/mpr.1954 (PMC10485316; doi:10.1002/mpr.1954)
Supplement: Supplementary file 1 — Supplementary Material [file MPR-32-e1954-s001.docx]

**Supplementary Materials**

**Measures and score calculations for additional outcomes described in Table 4**

***Screen for Disordered Eating (SDE)***

The Screen for Disordered Eating (SDE; Maguen et al., 2018) is comprised of five items to which respondents indicate whether they experience any disordered eating on a dichotomous scale (No=0 or Yes=1). In the primary care setting, this measure has demonstrated good discriminative accuracy (Maguen et al., 2018). In this study, we derived total scores by summing the five items resulting in a range from 0 to 5, with higher scores indicating higher levels of disordered eating.

***Insomnia Severity Index (ISI)***

The Insomnia Severity Index (ISI) is a psychometrically sound, 7-item self-report measure of insomnia symptoms over the previous two weeks (Bastien et al., 2001). Responses are reported on a Likert scale ranging from 0 (Not at all) to 4 (Very), producing total scores of 0 to 28. Cut-off scores are as follows: 0-7 reflects no clinically significant insomnia, 8-14 indicates subthreshold insomnia, 15-21 suggests moderate severity insomnia, and 22-28 indicates severe insomnia. The ISI was designed for use in adults but has been widely administered to, and validated in, adolescent samples (Alvaro et al., 2014; Chung et al., 2011).

***Pittsburgh Sleep Quality Index (PSQI)***

The Pittsburgh Sleep Quality Index (PSQI; Buysse et al., 1989) was designed to assesses sleep quality and disturbances over a 1-month interval. It consists of 19 items, which are used to compute seven component scores: sleep quality, sleep latency, sleep duration, habitual sleep efficiency, sleep disturbances, use of sleep medications and daytime dysfunction. Each item is weighted equally on a scale that ranges from 0 (no difficulty) to 3 (severe difficulty) scale. The seven component scores are then summed to yield a global score, ranging from 0 to 21; higher scores indicate worse sleep quality. The PSQI has demonstrated acceptable to good internal homogeneity, test-retest reliability and convergent validity across studies (Buysse et al., 1989; Hinz et al., 2017; Raniti et al., 2018).

***Strengths and Difficulties Questionnaire (SDQ)***

The Strengths and Difficulties Questionnaire (SDQ; Goodman, 1997) is a widely used behavioral screening questionnaire for 4 to 17 year-old children and adolescents. It consists of 25 items divided between 5 sub-scales: emotional symptoms, conduct problems, hyperactivity/inattention, peer relationship problems and prosocial behavior. Respondents indicate on a 3-point Likert scale the extent to which each item applies to them, using the options 0 (Not true), 1 (Somewhat true), or 2 (Certainly true). The Total Difficulties Score is comprised of all subscales except prosocial behavior and ranges from 0 to 40, with higher scores indicative of more problems. Internalizing (emotional symptoms and peer relationship problems) and Externalizing (conduct and hyperactivity problems) subscales each consist of 10-items with ranges from 0 to 20 (Goodman et al., 2010). The SDQ has demonstrated good internal consistency (Goodman, 2001; Muris et al., 2004).

***Short Warwick-Edinburgh Mental Wellbeing Scale (SWEMWBS)***

The Short Warwick-Edinburgh Mental Wellbeing Scale (SWEMWBS; Tennant et al., 2007) is a shortened 7-item version of the 14-item Warwick–Edinburgh Mental Well-being Scale (WEMWBS), which was developed to assess mental wellbeing in the general population. The SWEMWBS consists of seven statements about thoughts and feelings over the past two weeks. Ratings are made on a 5-point Likert scale (1=None of the time, 2=Rarely, 3=Some of the time, 4=Often, 5=All of the time). Total scale scores are calculated by summing item scores and transforming the total score using a conversion table. Total scores can range from 7 to 35. A higher score indicates a higher level of mental wellbeing. The SWEMWBS has demonstrated adequate reliability and validity (Haver et al., 2015; Vaingankar et al., 2017).

***Child Health Utility 9D (CHU-9D)***

The Child Health Utility 9D (CHU-9D; Stevens, 2009) is a 9 dimension generic preference-based measure designed to assess child and adolescent health related quality of life and suitable for application in economic evaluation. Current child/adolescent health related quality of life is assessed across the domains of worry, sadness, pain, tiredness, annoyance, school, sleep, daily routine and activities. Each dimension is rated on a 5-point response scale ranging from ‘no’ to ‘severe impairment’. Responses are then converted to utilities on the 0-1 dead to full health quality adjusted life years scale (QALY) using a preference weighted scoring algorithm (Ratcliffe et al., 2016). Previous validation studies with adolescents from the community and mental health services have demonstrated that the self-complete instrument has acceptable internal consistency and convergent validity for children and adolescents aged 7 to 17 years (Furber & Segal, 2015; Ratcliffe et al., 2012; Stevens & Ratcliffe, 2012).

**Measures and score calculations for outcomes described in Table 6**

***Ever self-harmed***

The Self-Harm Questionnaire (SHQ; Ougrin & Boege, 2013) was designed to improve identification of self-harm in adolescents. The complete questionnaire consists of three screening questions enquiring about any past incidents of self-harming behavior or thinking, followed by 12 additional questions that are only presented to adolescents reporting previous self-harm. To assess self-harm prevalence and frequency in the current study, only screening item 3 was administered to assess past episodes of self-harm (‘Have you ever actually harmed yourself on purpose? For example, have you ever cut yourself or taken an overdose and it was not an accident?’). Participants respond to this item on a 4-point scale of ‘No’, ‘Yes, once’, ‘Yes, two, three or four times’ and ‘Yes, five or more times’.

***Suicidal behavior in past 12 months***

The Youth Risk Behavior Survey (YRBS) was designed to assess health risk behaviors among secondary school students. Three items from the YRBS were used in the current study to assess suicide-related behaviors (thoughts, plans and attempts) in the past 12 months, for which participants indicate a ‘Yes’ or ‘No’ response. Studies have shown that the suicidality items demonstrate both substantial reliability (Brener et al., 2002) and good convergent and divergent validity in a secondary school sample (May & Klonsky, 2011).

***Psychotic symptoms***

The Adolescent Psychotic-Like Symptom Screener (APSS; Kelleher et al., 2011) is a 7-item measure designed to identify people who are at increased risk of future clinical psychotic disorder. In this study, only three items were administered to assess paranoia, auditory and visual hallucinations. For each question, there are 3 possible responses: ‘Yes, definitely’, ‘Maybe’, ‘No, never’. Frequency of ‘Yes, definitely’ responses to each item are shown in Table 6. In the general population, this instrument has demonstrated good sensitivity and specificity in identifying young adolescents with psychotic-like experiences (Kelleher et al., 2011).

***Alcohol use***

The alcohol use questionnaire included in this study was originally adapted from the School Health and Alcohol Harm Reduction Project (McBride et al., 2006) and used in the Climate Schools Projects (Newton et al., 2010), which are Australian school-based trials aimed at reducing alcohol and cannabis use. The questionnaire includes a standard drink diagram and contains items assessing age of first use, alongside frequency and quantity of alcohol use.

***Substance use in past 6 months***

A questionnaire assessing substance use was adapted from the Australian Institute of Health and Welfare 2007 National Drug Strategy Household Survey. The questionnaire contains items assessing substance use in the past 6 months, including cannabis, tobacco, amphetamine, ecstasy, hallucinogens, sedatives, inhalants, and other substance use.

**References**

Alvaro, P. K., Roberts, R. M., & Harris, J. K. (2014). The independent relationships between insomnia, depression, subtypes of anxiety, and chronotype during adolescence. *Sleep Med*, *15*(8), 934-941. https://doi.org/http://dx.doi.org/10.1016/j.sleep.2014.03.019

Bastien, C. H., Vallières, A., & Morin, C. M. (2001). Validation of the Insomnia Severity Index as an outcome measure for insomnia research. *Sleep Med*, *2*(4), 297-307.

Brener, N. D., Kann, L., McManus, T., Kinchen, S. A., Sundberg, E. C., & Ross, J. G. (2002). Reliability of the 1999 youth risk behavior survey questionnaire. *J Adolesc Health*, *31*(4), 336-342.

Buysse, D. J., Reynolds III, C. F., Monk, T. H., Berman, S. R., & Kupfer, D. J. (1989). The Pittsburgh Sleep Quality Index: A new instrument for psychiatric practice and research. *Psychiatry Res*, *28*(2), 193-213.

Chung, K. F., Kan, K. K.-K., & Yeung, W.-F. (2011). Assessing insomnia in adolescents: Comparison of insomnia severity index, athens insomnia scale and sleep quality index. *Sleep Med*, *12*(5), 463-470. http://ac.els-cdn.com/S1389945711001134/1-s2.0-S1389945711001134-main.pdf?_tid=a6215af6-afa4-11e6-8774-00000aacb362&acdnat=1479703417_600b23c8c0621cd32d3a50e44a52d9ba

Furber, G., & Segal, L. (2015). The validity of the Child Health Utility instrument (CHU9D) as a routine outcome measure for use in child and adolescent mental health services [journal article]. *Health Qual Life Outcomes*, *13*(1), 22. https://doi.org/10.1186/s12955-015-0218-4

Goodman, A., Lamping, D. L., & Ploubidis, G. B. (2010). When to use broader internalising and externalising subscales instead of the hypothesised five subscales on the Strengths and Difficulties Questionnaire (SDQ): data from British parents, teachers and children. *Journal of Abnormal Child Psychology*, *38*(8), 1179-1191.

Goodman, R. (1997). The Strengths and Difficulties Questionnaire: A Research Note. *J Child Psychol Psychiatry*, *38*(5), 581-586. https://doi.org/doi:10.1111/j.1469-7610.1997.tb01545.x

Goodman, R. (2001). Psychometric properties of the strengths and difficulties questionnaire. *J Am Acad Child Adolesc Psychiatry*, *40*(11), 1337-1345. https://doi.org/10.1097/00004583-200111000-00015

Haver, A., Akerjordet, K., Caputi, P., Furunes, T., & Magee, C. (2015). Measuring mental well-being: A validation of the Short Warwick–Edinburgh Mental Well-Being Scale in Norwegian and Swedish. *Scand J Public Health*, *43*(7), 721-727. https://doi.org/10.1177/1403494815588862

Hinz, A., Glaesmer, H., Brähler, E., Löffler, M., Engel, C., Enzenbach, C., Hegerl, U., & Sander, C. (2017). Sleep quality in the general population: psychometric properties of the Pittsburgh Sleep Quality Index, derived from a German community sample of 9284 people. *Sleep Med*, *30*, 57-63. https://doi.org/https://doi.org/10.1016/j.sleep.2016.03.008

Kelleher, I., Harley, M., Murtagh, A., & Cannon, M. (2011). Are screening instruments valid for psychotic-like experiences? A validation study of screening questions for psychotic-like experiences using in-depth clinical interview. *Schizophr Bull*, *37*(2), 362-369. https://doi.org/10.1093/schbul/sbp057

Maguen, S., Hebenstreit, C., Li, Y., Dinh, J. V., Donalson, R., Dalton, S., Rubin, E., & Masheb, R. (2018). Screen for Disordered Eating: Improving the accuracy of eating disorder screening in primary care. *Gen Hosp Psychiatry*, *50*, 20-25. https://doi.org/https://doi.org/10.1016/j.genhosppsych.2017.09.004

May, A., & Klonsky, E. D. (2011). Validity of suicidality items from the Youth Risk Behavior Survey in a high school sample. *Assessment*, *18*(3), 379-381.

McBride, N., National Drug Research Institute (Australia), McBride, N., Farringdon, F., Midford, R., & Muleners, L. (2006). *School Health and Alcohol Harm Reduction Project: Details of intervention development and research procedures*. National Drug Research Institute, Curtin University of Technology.

Muris, P., Meesters, C., Eijkelenboom, A., & Vincken, M. (2004). The self-report version of the Strengths and Difficulties Questionnaire: Its psychometric properties in 8- to 13-year-old non-clinical children. *Br J Clin Psychol*, *43*(Pt 4), 437-448. https://doi.org/10.1348/0144665042388982

Newton, N. C., Teesson, M., Vogl, L. E., & Andrews, G. (2010). Internet-based prevention for alcohol and cannabis use: Final results of the Climate Schools course. *Addiction*, *105*(4), 749-759. https://doi.org/10.1111/j.1360-0443.2009.02853.x

Ougrin, D., & Boege, I. (2013). Brief report: The self harm questionnaire: A new tool designed to improve identification of self harm in adolescents. *J Adolesc*, *36*(1), 221-225. https://doi.org/https://doi.org/10.1016/j.adolescence.2012.09.006

Raniti, M. B., Waloszek, J. M., Schwartz, O., Allen, N. B., & Trinder, J. (2018). Factor structure and psychometric properties of the Pittsburgh Sleep Quality Index in community-based adolescents. *Sleep*, *41*(6), zsy066. https://doi.org/10.1093/sleep/zsy066

Ratcliffe, J., Flynn, T., Terlich, F., Stevens, K., Brazier, J., & Sawyer, M. (2012). Developing adolescent-specific health state values for economic evaluation: An application of profile case best-worst scaling to the Child Health Utility 9D. *Pharmacoeconomics*, *30*(8), 713-727. https://doi.org/10.2165/11597900-000000000-00000

Ratcliffe, J., Huynh, E., Chen, G., Stevens, K., Swait, J., Brazier, J., Sawyer, M., Roberts, R., & Flynn, T. (2016). Valuing the Child Health Utility 9D: Using profile case best worst scaling methods to develop a new adolescent specific scoring algorithm. *Soc Sci Med*, *157*, 48-59. https://doi.org/10.1016/j.socscimed.2016.03.042

Stevens, K. (2009). Developing a descriptive system for a new preference-based measure of health-related quality of life for children. *Qual Life Res*, *18*(8), 1105-1113. https://doi.org/10.1007/s11136-009-9524-9

Stevens, K., & Ratcliffe, J. (2012). Measuring and Valuing Health Benefits for Economic Evaluation in Adolescence: An Assessment of the Practicality and Validity of the Child Health Utility 9D in the Australian Adolescent Population. *Value Health*, *15*(8), 1092-1099. https://doi.org/https://doi.org/10.1016/j.jval.2012.07.011

Tennant, R., Hiller, L., Fishwick, R., Platt, S., Joseph, S., Weich, S., Parkinson, J., Secker, J., & Stewart-Brown, S. (2007). The Warwick-Edinburgh Mental Well-being Scale (WEMWBS): Development and UK validation [journal article]. *Health Qual Life Outcomes*, *5*(1), 63. https://doi.org/10.1186/1477-7525-5-63

Vaingankar, J. A., Abdin, E., Chong, S. A., Sambasivam, R., Seow, E., Jeyagurunathan, A., Picco, L., Stewart-Brown, S., & Subramaniam, M. (2017). Psychometric properties of the short Warwick Edinburgh mental well-being scale (SWEMWBS) in service users with schizophrenia, depression and anxiety spectrum disorders [journal article]. *Health Qual Life Outcomes*, *15*(1), 153. https://doi.org/10.1186/s12955-017-0728-3
